# Cardiac Complications After SARS-CoV-2 Infection and mRNA COVID-19 Vaccination — PCORnet, United States, January 2021–January 2022

**DOI:** 10.15585/mmwr.mm7114e1

**Published:** 2022-04-08

**Authors:** Jason P. Block, Tegan K. Boehmer, Christopher B. Forrest, Thomas W. Carton, Grace M. Lee, Umed A. Ajani, Dimitri A. Christakis, Lindsay G. Cowell, Christine Draper, Nidhi Ghildayal, Aaron M. Harris, Michael D. Kappelman, Jean Y. Ko, Kenneth H. Mayer, Kshema Nagavedu, Matthew E. Oster, Anuradha Paranjape, Jon Puro, Matthew D. Ritchey, David K. Shay, Deepika Thacker, Adi V. Gundlapalli

**Affiliations:** ^1^Department of Population Medicine, Harvard Pilgrim Health Care Institute, Harvard Medical School, Boston, Massachusetts; ^2^CDC COVID-19 Emergency Response Team; ^3^Applied Clinical Research Center, Department of Pediatrics, Children’s Hospital of Philadelphia, Philadelphia, Pennsylvania; ^4^Louisiana Public Health Institute, New Orleans, Louisiana; ^5^Department of Pediatrics, Stanford University School of Medicine, Stanford, California; ^6^Center for Child Health, Behavior and Development, Seattle Children’s Research Institute, Seattle Children’s Hospital, Seattle, Washington; ^7^Department of Population and Data Sciences and Department of Immunology, University of Texas Southwestern Medical Center, Dallas, Texas; ^8^Center for Gastrointestinal Biology and Disease, University of North Carolina School of Medicine, Chapel Hill, North Carolina; ^9^The Fenway Institute, Fenway Health, Harvard Medical School, Boston, Massachusetts; ^10^Children’s Healthcare of Atlanta, Emory University School of Medicine, Atlanta, Georgia; ^11^Department of Medicine, Lewis Katz School of Medicine at Temple University, Philadelphia, Pennsylvania; ^12^OCHIN, Inc., Portland, Oregon; ^13^Nemours Cardiac Center, Nemours Children’s Health System, Wilmington, Delaware.

Cardiac complications, particularly myocarditis and pericarditis, have been associated with SARS-CoV-2 (the virus that causes COVID-19) infection (*1*–*3*) and mRNA COVID-19 vaccination (*2*–*5*). Multisystem inflammatory syndrome (MIS) is a rare but serious complication of SARS-CoV-2 infection with frequent cardiac involvement (*6*). Using electronic health record (EHR) data from 40 U.S. health care systems during January 1, 2021–January 31, 2022, investigators calculated incidences of cardiac outcomes (myocarditis; myocarditis or pericarditis; and myocarditis, pericarditis, or MIS) among persons aged ≥5 years who had SARS-CoV-2 infection, stratified by sex (male or female) and age group (5–11, 12–17, 18–29, and ≥30 years). Incidences of myocarditis and myocarditis or pericarditis were calculated after first, second, unspecified, or any (first, second, or unspecified) dose of mRNA COVID-19 (BNT162b2 [Pfizer-BioNTech] or mRNA-1273 [Moderna]) vaccines, stratified by sex and age group. Risk ratios (RR) were calculated to compare risk for cardiac outcomes after SARS-CoV-2 infection to that after mRNA COVID-19 vaccination. The incidence of cardiac outcomes after mRNA COVID-19 vaccination was highest for males aged 12–17 years after the second vaccine dose; however, within this demographic group, the risk for cardiac outcomes was 1.8–5.6 times as high after SARS-CoV-2 infection than after the second vaccine dose. The risk for cardiac outcomes was likewise significantly higher after SARS-CoV-2 infection than after first, second, or unspecified dose of mRNA COVID-19 vaccination for all other groups by sex and age (RR 2.2–115.2). These findings support continued use of mRNA COVID-19 vaccines among all eligible persons aged ≥5 years.

This study used EHR data from 40 health care systems* participating in PCORnet, the National Patient-Centered Clinical Research Network (*7*), during January 1, 2021–January 31, 2022. PCORnet is a national network of networks that facilitates access to health care data and interoperability through use of a common data model across participating health care systems (https://pcornet.org/data). The PCORnet Common Data Model contains information captured from EHRs and other health care data sources (e.g., health insurance claims), including demographic characteristics, diagnoses, prescriptions, procedures, and laboratory test results, among other elements. The study population included persons with documented SARS-CoV-2 testing, viral illness diagnostic codes, or COVID-19 vaccination during the study period. Data were obtained through a single query that was executed by participating health care systems to generate aggregated results.

Five cohorts were created using coded EHR data among persons aged ≥5 years: 1) an infection cohort (persons who received ≥1 positive SARS-CoV-2 molecular or antigen test result); 2) a first dose cohort (persons who received a first dose of an mRNA COVID-19 vaccine); 3) a second dose cohort (persons who received a second dose of an mRNA COVID-19 vaccine); 4) an unspecified dose cohort (persons who received an mRNA COVID-19 vaccine dose not specified as a first or second dose); and 5) an any dose cohort (persons who received any mRNA COVID-19 vaccine dose). The any dose cohort is a combination of the other three vaccination cohorts; persons who received 2 doses were included twice in this cohort, once for each dose.^†^ Vaccine doses specifically coded as booster or extra doses were excluded. Persons with a positive SARS-CoV-2 test result ≤30 days before receipt of an mRNA COVID-19 vaccine were excluded from the vaccine cohorts; persons who had received an mRNA COVID-19 vaccine dose ≤30 days before a positive SARS-CoV-2 test result were excluded from the infection cohort. In the infection cohort, there were no other exclusions based on vaccination status. The following index dates were used for cohort entrance: first positive SARS-CoV-2 test result for the infection cohort; first vaccination for the first dose cohort; second vaccination for the second dose cohort; the single vaccination for the unspecific dose cohort; and the first, second, and unspecified vaccination for the any dose cohort. Persons could be represented twice in the any dose cohort if they received a first and second dose; they would have a different index date for each of the doses.

Incidence of three cardiac outcomes (myocarditis; myocarditis or pericarditis; and myocarditis, pericarditis, or MIS) were defined using *International Classification of Diseases, Tenth Revision, Clinical Modification* (ICD-10-CM) diagnostic codes^§^ within 7-day or 21-day risk windows after the index date; persons who had received any of these diagnoses during the year preceding the index date were excluded. The outcome including MIS was only assessed for the infection cohort because the rare reports of MIS after mRNA COVID-19 vaccination typically had evidence of previous SARS-CoV-2 infection (*8*); a 42-day risk window also was used for this outcome to allow for a possible long latency between infection and diagnosis of MIS (*6*).^¶^ Because persons with MIS who have cardiac involvement might only receive an ICD-10-CM code for MIS, rather than myocarditis or pericarditis, this combined outcome allowed for a comprehensive capture of potential cardiac complications after infection. Nearly 80% of cases of MIS have cardiac involvement (*9*). Cohorts were stratified by sex and age group.

The sex- and age-stratified incidences of the cardiac outcomes (cases per 100,000 persons) were calculated within 7-, 21-, or 42-day risk windows. Unadjusted RRs and 95% CIs were calculated as the incidences of the outcomes within the infection cohort divided by the incidences in the first, second, unspecified, and any dose cohorts separately for each sex and age stratum. RRs whose CIs did not include 1.0 were considered statistically significant; RRs were not compared across outcomes, risk windows, vaccine dose, or sex and age stratum. This activity was reviewed by CDC and was conducted consistent with applicable federal law and CDC policy.**

The study population consisted of 15,215,178 persons aged ≥5 years, including 814,524 in the infection cohort; 2,548,334 in the first dose cohort; 2,483,597 in the second dose cohort; 1,681,169 in the unspecified dose cohort; and 6,713,100 in the any dose cohort (Table 1).^††^ Among the four COVID-19 vaccination cohorts, 77%–79% of persons were aged ≥30 years; within the SARS-CoV-2 infection cohort, 63% were aged ≥30 years.

**TABLE 1:** Demographic characteristics of persons who were infected with SARS-CoV-2 or received a first, second, unspecified, or any dose of an mRNA COVID-19 vaccine* — National Patient-Centered Clinical Research Network, United States, January 1, 2021–January 31, 2022

Characteristic	No. (%)				
	SARS-CoV-2 infection cohort^†^	mRNA COVID-19 vaccination cohort			
		First dose*^,§^	Second dose*^,§^	Unspecified dose*^,¶^	Any dose*^,^**
**Cohort total**	**814,524 (100)**	**2,548,334 (100)**	**2,483,597 (100)**	**1,681,169 (100)**	**6,713,100 (100)**
**Age group, yrs**					
5–11	76,960 (9)	48,986 (2)	41,742 (2)	30,199 (2)	120,927 (2)
12–17	70,336 (9)	190,810 (7)	179,612 (7)	113,775 (7)	484,197 (7)
18–29	151,950 (19)	308,892 (12)	297,560 (12)	241,787 (14)	848,239 (13)
30–50	255,103 (31)	665,876 (26)	652,947 (26)	490,808 (29)	1,809,631 (27)
51–65	152,243 (19)	601,615 (24)	588,873 (24)	404,445 (24)	1,594,933 (24)
≥66	107,932 (13)	732,155 (29)	722,863 (29)	400,155 (24)	1,855,173 (28)
**Sex**					
Female	457,506 (56)	1,497,984 (59)	1,463,746 (59)	997,741 (59)	3,959,471 (59)
Male	357,018 (44)	1,050,350 (41)	1,019,851 (41)	683,428 (41)	2,753,629 (41)
**Race/Ethnicity** ^††^					
Hispanic	133,784 (16)	309,468 (12)	298,270 (12)	169,688 (10)	777,426 (12)
Asian	23,684 (3)	133,445 (5)	131,205 (5)	83,937 (5)	348,587 (5)
Black or African American	162,434 (20)	408,657 (16)	395,283 (16)	283,534 (17)	1,087,474 (16)
Other	34,473 (4)	93,100 (4)	90,122 (4)	54,305 (3)	237,527 (4)
White	408,152 (50)	1,441,573 (57)	1,407,974 (57)	1,001,686 (60)	3,851,233 (57)
Missing^§§^	58,980 (7)	205,834 (8)	204,224 (8)	98,299 (6)	508,357 (8)

* In the first and second dose cohorts, 27% of persons received mRNA-1273 (Moderna) vaccine and 73% received BNT162b2 (Pfizer-BioNTech) vaccine. In the unspecified dose cohort, 36% received Moderna and 64% Pfizer-BioNTech. In the any dose cohort, 29% received Moderna and 71% Pfizer-BioNTech.

^†^ Persons in the infection cohort included those who received ≥1 positive SARS-CoV-2 molecular or antigen test result.

^§^ The first dose cohort included persons who had either the first of 2 doses ≥20 days before a second dose or a specific code for a first dose; the second dose cohort included persons who had either the second of 2 doses ≥20 days after a first dose or a specific code for a second dose.

^¶^ The unspecified dose cohort included persons who had a single dose that was not specified as a first or second dose; doses specified as booster doses were excluded.

** The any dose cohort is the first, second, and unspecified dose cohorts combined; persons who had 2 doses are represented twice in the cohort but had different index dates for their first and second doses.

^††^ Persons of Hispanic origin could be of any race; Asian, Black or African American, White, or other (which includes American Indian or Alaska Native, Native Hawaiian or Other Pacific Islander, multiple race, and other race) persons are not Hispanic.

^§§^ Missing race category includes no information, refused to answer, unknown, or missing.

Among males aged 5–11 years, the incidences of myocarditis and myocarditis or pericarditis were 12.6–17.6 cases per 100,000 after infection, 0–4 after the first vaccine dose, and 0 after the second dose; incidences of myocarditis, pericarditis, or MIS were 93.0–133.2 after infection (Table 2). Because there were no or few cases of myocarditis or pericarditis after vaccination, the RRs for several comparisons could not be calculated or were not statistically significant. The RRs were significant when comparing myocarditis, pericarditis, or MIS in the 42 days after infection (133.2 cases per 100,000) with myocarditis or pericarditis after the first (4.0 cases per 100,000; RR 33.3) or second (4.7 cases per 100,000; RR 28.2) vaccine dose.

**TABLE 2:** Incidence of cardiac outcomes among males aged ≥5 years after SARS-CoV-2 infection or mRNA COVID-19 vaccination and risk ratios, by age group and risk window — National Patient-Centered Clinical Research Network, United States, January 1, 2021–January 31, 2022

Age group, yrs/Outcome/Risk window	Incidence* among males					Risk ratio (95% CI) SARS-CoV-2 infection versus mRNA COVID-19 vaccination			
	SARS-CoV-2 infection cohort^†^	mRNA COVID-19 vaccination cohort				mRNA COVID-19 vaccination cohort			
		First dose^§^	Second dose^§^	Unspecified dose^¶^	Any dose**	First dose^§^	Second dose^§^	Unspecified dose^¶^	Any dose**
**5–11^††^**									
**Myocarditis**									
7-day	12.6	0	0	0	0	NC	NC	NC	NC
21-day	17.6	4.0	0	6.5	3.2	4.4 (0.5–35.7)	NC	2.7 (0.3–22.1)	5.4 (1.1–26.1)
**Myocarditis or pericarditis**									
7-day	12.6	0	0	0	0	NC	NC	NC	NC
21-day	17.6	4.0	0	6.5	3.2	4.4 (0.5–35.7)	NC	2.7 (0.3–22.1)	5.4 (1.1–26.1)
**Myocarditis, pericarditis, or MIS** ^§§^									
7-day	93.0	—^¶¶^	—	—	—	NC	NC	NC	NC
21-day	103.0	—	—	—	—	25.7 (3.5–187.0)	NC	16.0 (2.2–116.0)	31.7 (7.7–131.2)
42-day	133.2	—	—	—	—	33.3 (4.6–240.5)	28.2 (3.9–203.9)	10.3 (2.5–42.3)	20.5 (7.4–56.7)
**12–17** ^††^									
**Myocarditis**									
7-day	50.1	2.2	22.0	16.7	12.9	23.0 (5.3–99.5)	2.3 (1.2–4.4)	3.0 (1.3–6.7)	3.9 (2.1–7.0)
21-day	59.0	3.3	26.7	20.4	16.0	18.0 (5.4–60.6)	2.2 (1.2–4.0)	2.9 (1.4–6.0)	3.7 (2.1–6.4)
**Myocarditis or pericarditis**									
7-day	56.0	2.2	26.7	22.3	16.0	25.7 (6.0–110.3)	2.1 (1.1–3.9)	2.5 (1.2–5.2)	3.5 (2.0–6.1)
21-day	64.9	3.3	35.9	29.7	21.6	19.8 (5.9–66.2)	1.8 (1.0–3.1)	2.2 (1.1–4.2)	3.0 (1.8–5.0)
**Myocarditis, pericarditis, or MIS** ^§§^									
7-day	150.5	—	—	—	—	69.0 (16.8–283.2)	5.6 (3.5–9.2)	6.8 (3.6–12.7)	9.4 (6.2–14.4)
21-day	159.3	—	—	—	—	48.7 (15.2–155.7)	4.4 (2.9–6.9)	5.4 (3.1–9.4)	7.4 (5.0–10.8)
42-day	180.0	—	—	—	—	4.9 (3.2–7.4)	4.6 (3.0–6.9)	5.4 (3.2–9.1)	4.9 (3.5–6.7)
**18–29**									
**Myocarditis**									
7-day	55.3	0.9	6.5	7.0	4.6	61.8 (8.5–451.8)	8.5 (3.7–19.1)	7.9 (3.3–19.0)	12.0 (6.4–22.5)
21-day	63.7	3.6	8.4	11.6	7.5	17.8 (6.4–49.8)	7.6 (3.7–15.7)	5.5 (2.7–11.0)	8.4 (5.0–14.2)
**Myocarditis or pericarditis**									
7-day	85.5	2.7	12.1	22.0	11.5	31.8 (9.9–102.0)	7.0 (3.8–12.9)	3.9 (2.3–6.6)	7.4 (4.8–11.5)
21-day	100.6	8.1	15.0	27.8	16.1	12.5 (6.2–25.2)	6.7 (3.9–11.7)	3.6 (2.3–5.8)	6.3 (4.3–9.1)
**Myocarditis, pericarditis, or MIS** ^§§^									
7-day	97.2	—	—	—	—	36.2 (11.3–115.5)	8.0 (4.4–14.6)	4.4 (2.6–7.4)	8.5 (5.6–12.9)
21-day	112.3	—	—	—	—	13.9 (7.0–28.0)	7.5 (4.4–13.0)	4.0 (2.5–6.4)	7.0 (4.8–10.1)
42-day	140.8	—	—	—	—	7.2 (4.5–11.4)	8.4 (5.0–13.9)	4.5 (2.9–6.9)	6.4 (4.6–8.8)
**≥30**									
**Myocarditis**									
7-day	57.2	0.9	0.5	3.0	1.3	67.2 (31.4–143.8)	115.2 (42.6–311.7)	18.9 (11.2–31.7)	45.7 (30.2–69.2)
21-day	63.0	1.9	1.2	4.2	2.2	32.4 (19.3–54.3)	50.8 (26.7–96.4)	15.1 (9.7–23.7)	28.3 (20.4–39.3)
**Myocarditis or pericarditis**									
7-day	100.2	3.8	3.1	15.0	6.3	26.6 (18.2–38.7)	32.3 (21.3–48.8)	6.7 (5.2–8.6)	16.0 (12.9–19.8)
21-day	114.0	7.3	7.3	20.1	10.4	15.6 (11.8–20.7)	15.6 (11.7–20.7)	5.7 (4.5–7.1)	10.9 (9.1–13.1)
**Myocarditis, pericarditis, or MIS** ^§§^									
7-day	109.1	—	—	—	—	28.9 (19.9–42.0)	35.1 (23.3–53.0)	7.3 (5.7–9.4)	17.4 (14.1–21.5)
21-day	123.0	—	—	—	—	16.8 (12.7–22.3)	16.8 (12.7–22.2)	6.1 (4.9–7.7)	11.8 (9.9–14.0)
42-day	136.8	—	—	—	—	10.7 (8.6–13.4)	10.8 (8.6–13.5)	5.4 (4.4–6.7)	8.7 (7.4–10.1)

**Abbreviations**: MIS = multisystem inflammatory syndrome; NC = not calculated.

* Cases per 100,000 persons.

^†^ Persons in the infection cohort included those who received ≥1 positive SARS-CoV-2 molecular or antigen test result.

^§^ The first dose cohort included persons who had either the first of 2 doses ≥20 days before a second dose or a specific code for a first dose; the second dose cohort included persons who had either the second of 2 doses ≥20 days after a first dose or a specific code for a second dose.

^¶^ The unspecified dose cohort included persons who had a single dose that was not specified as a first or second dose; doses specified as booster doses were excluded.

** The any dose cohort is the first, second, and unspecified dose cohorts combined; persons who had 2 doses are represented twice in the cohort but had different index dates for their first and second doses.

^††^ BNT162b2 (Pfizer-BioNTech) is the only mRNA COVID-19 vaccine approved for persons aged 5–17 years.

^§§^ Diagnoses of myocarditis, pericarditis, or MIS after a positive SARS-CoV-2 test result compared with diagnoses of myocarditis or pericarditis after vaccination. The 42-day risk ratios were only calculated for this outcome and comparison. The incidence of myocarditis or pericarditis in this risk window was 4.0, 37.1, 19.7, and 12.8 cases per 100,000 for males aged 5–11, 12–17, 18–29, and ≥30 years after a first dose of an mRNA COVID-19 vaccine; 4.7, 39.4, 16.8, and 12.7 cases per 100,000 after a second dose; 12.9, 33.4, 31.3, and 25.3 cases per 100,000 after an unspecified dose; and 6.5, 37.1, 22.0, and 15.8 cases per 100,000 after any dose.

^¶¶^ Dashes indicate the incidence for vaccination cohorts was not applicable because the comparison for incidence of myocarditis, pericarditis, or MIS after infection was to myocarditis or pericarditis after vaccination.

Among males aged 12–17 years, the incidences of myocarditis and myocarditis or pericarditis were 50.1–64.9 cases per 100,000 after infection, 2.2–3.3 after the first vaccine dose, and 22.0–35.9 after the second dose; incidences of myocarditis, pericarditis, or MIS were 150.5–180.0 after infection. RRs for cardiac outcomes comparing infected persons with first dose recipients were 4.9–69.0, and with second dose recipients, were 1.8–5.6; all RRs were statistically significant.

Among males aged 18–29 years, the incidences of myocarditis and myocarditis or pericarditis were 55.3–100.6 cases per 100,000 after infection, 0.9–8.1 after the first vaccine dose, and 6.5–15.0 after the second dose; incidences of myocarditis, pericarditis, or MIS were 97.2–140.8 after infection. RRs for cardiac outcomes comparing infected persons with first dose recipients were 7.2–61.8, and with second dose recipients, were 6.7–8.5; all RRs were statistically significant.

Among males aged ≥30 years, the incidences of myocarditis and myocarditis or pericarditis were 57.2–114.0 cases per 100,000 after infection, 0.9–7.3 after the first vaccine dose, and 0.5–7.3 after the second dose; incidences of myocarditis, pericarditis, or MIS were 109.1–136.8 after infection. RRs for cardiac outcomes among infected persons compared with first dose recipients were 10.7–67.2, and compared with second dose recipients, were 10.8–115.2; all RRs were statistically significant.

Among females aged 5–11 years, incidences of myocarditis and myocarditis or pericarditis were 5.4–10.8 cases per 100,000 after infection, and incidences of myocarditis, pericarditis, or MIS were 67.3–94.2 after infection (Table 3). No cases of myocarditis or pericarditis after vaccination were identified. The incidences of cardiac outcomes did not vary by age among females aged ≥12 years. In this group, the incidences of myocarditis and myocarditis or pericarditis were 11.9–61.7 cases per 100,000 after infection, 0.5–6.2 after the first vaccine dose, and 0.5–5.4 after the second dose; incidences of myocarditis, pericarditis, or MIS were 27.1–93.3 after infection. Among females aged ≥12 years, RRs for cardiac outcomes comparing infected persons with first dose recipients were 7.4–42.6, and with second dose recipients, were 6.4–62.9; all RRs were statistically significant.

**TABLE 3:** Incidence of cardiac outcomes among females aged ≥5 years after SARS-CoV-2 infection or mRNA COVID-19 vaccination and risk ratios, by age group and risk window — National Patient-Centered Clinical Research Network, United States, January 1, 2021–January 31, 2022

Age group, yrs/Outcome/Risk window	Incidence* among females					Risk ratio (95% CI) SARS-CoV-2 infection versus mRNA COVID-19 vaccination			
	SARS-CoV-2 infection cohort^†^	mRNA COVID-19 vaccination cohort				mRNA COVID-19 vaccination cohort			
		First dose^§^	Second dose^§^	Unspecified dose^¶^	Any dose**	First dose^§^	Second dose^§^	Unspecified dose^¶^	Any dose**
**5–11** ^††^									
**Myocarditis**									
7-day	5.4	0	0	0	0	NC	NC	NC	NC
21-day	8.1	0	0	0	0	NC	NC	NC	NC
**Myocarditis or pericarditis**									
7-day	8.1	0	0	0	0	NC	NC	NC	NC
21-day	10.8	0	0	0	0	NC	NC	NC	NC
**Myocarditis, pericarditis, or MIS** ^§§^									
7-day	67.3	—^¶¶^	—	—	—	NC	NC	NC	NC
21-day	80.7	—	—	—	—	NC	NC	NC	NC
42-day	94.2	—	—	—	—	NC	NC	NC	NC
**12–17** ^††^									
**Myocarditis**									
7-day	24.7	1.0	1.1	0	0.8	24.5 (3.1–193.3)	23.1 (2.9–182.0)	NC	31.2 (6.7–144.3)
21-day	35.7	1.0	3.2	1.7	2.0	35.4 (4.6–270.5)	11.1 (3.2–39.0)	21.4 (2.8–163.4)	18.0 (6.4–50.5)
**Myocarditis or pericarditis**									
7-day	24.7	2.0	2.1	0	1.6	12.2 (2.6–56.7)	11.5 (2.5–53.4)	NC	15.6 (4.8–50.6)
21-day	35.7	2.0	5.4	3.3	3.6	17.7 (4.0–78.4)	6.7 (2.4–18.7)	10.7 (2.4–47.4)	10.0 (4.3–23.4)
**Myocarditis, pericarditis, or MIS** ^§§^									
7-day	63.1	—	—	—	—	31.3 (7.4–132.7)	29.5 (6.9–125.0)	NC	39.8 (13.8–115.2)
21-day	79.6	—	—	—	—	39.5 (9.4–165.4)	14.9 (5.7–38.3)	23.8 (5.7–99.9)	22.3 (10.6–47.2)
42-day	93.3	—	—	—	—	11.6 (5.4–25.0)	12.4 (5.5–28.1)	14.0 (5.0–39.4)	12.4 (7.1–21.7)
**18–29**									
**Myocarditis**									
7-day	11.9	0.5	1.6	3.9	1.8	23.5 (3.0–182.0)	7.6 (2.1–27.1)	3.1 (1.1–8.4)	6.5 (2.8–15.2)
21-day	19.5	1.0	2.1	5.8	2.8	19.2 (4.5–82.9)	9.3 (3.1–27.5)	3.4 (1.5–7.5)	7.1 (3.6–14.0)
**Myocarditis or pericarditis**									
7-day	23.8	2.5	3.1	7.1	4.0	9.4 (3.6–24.8)	7.6 (3.1–18.7)	3.4 (1.6–7.0)	5.9 (3.3–10.6)
21-day	33.6	4.6	5.2	10.9	6.6	7.4 (3.5–15.5)	6.4 (3.1–13.1)	3.1 (1.7–5.6)	5.1 (3.1–8.2)
**Myocarditis, pericarditis, or MIS** ^§§^									
7-day	27.1	—	—	—	—	10.7 (4.1–27.9)	8.6 (3.5–21.0)	3.8 (1.9–7.8)	6.7 (3.8–11.9)
21-day	40.1	—	—	—	—	8.8 (4.2–18.2)	7.6 (3.8–15.4)	3.7 (2.1–6.5)	6.1 (3.8–9.6)
42-day	67.2	—	—	—	—	8.3 (4.8–14.3)	11.6 (6.1–22.1)	5.2 (3.2–8.7)	7.8 (5.3–11.3)
**≥30**									
**Myocarditis**									
7-day	32.6	0.8	0.5	1.0	0.7	42.6 (21.5–84.4)	62.9 (27.6–143.6)	31.3 (15.2–64.3)	44.0 (27.9–69.3)
21-day	36.3	1.4	0.9	1.6	1.3	25.2 (15.1–42.0)	38.3 (20.6–71.3)	23.2 (12.8–42.2)	28.2 (19.6–40.6)
**Myocarditis or pericarditis**									
7-day	53.8	3.1	1.7	8.2	3.9	17.1 (12.0–24.5)	31.2 (19.6–49.7)	6.6 (4.9–8.8)	13.9 (11.0–17.7)
21-day	61.7	6.2	4.1	10.7	6.5	10.0 (7.6–13.1)	14.9 (10.8–20.5)	5.8 (4.5–7.5)	9.4 (7.7–11.5)
**Myocarditis, pericarditis, or MIS** ^§§^									
7-day	58.6	—	—	—	—	18.7 (13.1–26.6)	34.0 (21.4–54.0)	7.1 (5.4–9.5)	15.2 (12.0–19.2)
21-day	68.2	—	—	—	—	11.0 (8.4–14.4)	16.5 (12.0–22.6)	6.4 (4.9–8.3)	10.4 (8.6–12.7)
42-day	79.6	—	—	—	—	8.4 (6.7–10.5)	10.0 (7.9–12.8)	5.6 (4.5–7.0)	7.9 (6.7–9.4)

**Abbreviations**: MIS = multisystem inflammatory syndrome; NC = not calculated.

* Cases per 100,000 persons.

^†^ Persons in the infection cohort included those who received ≥1 positive SARS-CoV-2 molecular or antigen test result.

^§^ The first dose cohort included persons who had either the first of 2 doses ≥20 days before a second dose or a specific code for a first dose; the second dose cohort included persons who had either the second of 2 doses ≥20 days after a first dose or a specific code for a second dose.

^¶^ The unspecified dose cohort included persons who had a single dose that was not specified as a first or second dose; doses specified as booster doses were excluded.

** The any dose cohort is the first, second, and unspecified dose cohorts combined; persons who had 2 doses are represented twice in the cohort but had different index dates for their first and second doses.

^††^ BNT162b2 (Pfizer-BioNTech) is the only mRNA COVID-19 vaccine approved for persons aged 5–17 years.

^§§^ Diagnoses of myocarditis, pericarditis, or MIS after a positive SARS-CoV-2 test result compared with diagnoses of myocarditis or pericarditis after vaccination. The 42-day risk ratios were only calculated for this outcome and comparison. The incidence of myocarditis or pericarditis in this risk window was 0, 8.1, 8.1, 9.5 cases per 100,000 for females 5-11, 12-17, 18-29, and ≥30 years after a first dose of an mRNA COVID-19 vaccine; 0, 7.5, 5.8, and 8.0 cases per 100,000 after a second dose; 0, 6.7, 12.9, and 14.2 cases per 100,000 after an unspecified dose; and 0, 7.5, 8.7, and 10.1 cases per 100,000 after any dose.

^¶¶^ Dashes indicate the incidence for vaccination cohorts was not applicable because the comparison for incidence of myocarditis, pericarditis, or MIS after infection was to myocarditis or pericarditis after vaccination.

## Discussion

Analysis of EHR data from 40 U.S. health care systems found that the incidences of cardiac complications after SARS-CoV-2 infection or mRNA COVID-19 vaccination were low overall but were higher after infection than after vaccination for both males and females in all age groups. Two studies from Israel (*2*) and the United Kingdom (*3*) have found similar higher risk for myocarditis after SARS-CoV-2 infection compared with that after mRNA COVID-19 vaccination.

Myocarditis or pericarditis incidence after mRNA COVID-19 vaccination in the current study (0–35.9 per 100,000 for males and 0–10.9 for females across age groups and vaccine cohorts) was similar to estimates found in a study from eight U.S. health systems in the Vaccine Safety Datalink (*10*). Previous CDC estimates found the highest risk for post-vaccination myocarditis among males aged 16–17 years (10.6 per 100,000) during a 7-day risk window after receipt of a second mRNA COVID-19 vaccine dose (*5*). Estimates from the current study (22.0 per 100,000 males aged 12–17 years) are higher, likely because outcomes were captured using ICD-10-CM codes alone rather than through passive reporting with subsequent verification through medical record review. Even among males aged 12–17 years, the group with the highest incidence of cardiac complications after receipt of a second mRNA COVID-19 vaccine dose, the risk was 1.8–5.6 times as high after SARS-CoV-2 infection than after vaccination.

The findings in this report are subject to at least six limitations. First, data were obtained using a query that returned aggregate data from sites, precluding adjustment for potential confounders. Stratification by age and sex was performed because of their clear prior association with cardiac outcomes. Second, outcomes were rare in some cohorts, leading to wide CIs around RR estimates. Third, only SARS-CoV-2 test results and mRNA COVID-19 vaccinations documented in EHRs were available for assessment. SARS-CoV-2 infections were not captured if testing occurred in homes, schools, community sites, or pharmacies. Similarly, EHR data in this study captured ≥1 dose of mRNA COVID-19 vaccine for 28% of persons aged ≥5 years. Nationally, 82% of persons aged ≥5 years were reported to have received any COVID-19 vaccination; 97% of all vaccinations administered were mRNA COVID-19 vaccines.^§§^ Underascertainment of SARS-CoV-2 infections and mRNA COVID-19 vaccinations reduced sample size and might have introduced bias if capture of infection or vaccination within the EHR occurred differentially for those with cardiac outcomes.^¶¶^ Fourth, case definitions for myocarditis, pericarditis, or MIS were ICD-10-CM code–based; diagnoses were not confirmed with chart review and are subject to misclassification. Fifth, cases of MIS among persons without documented SARS-CoV-2 infection were not included (*9*). Finally, some overlap might have occurred in risk windows for persons who had a SARS-CoV-2 infection soon after vaccination or a vaccination soon after infection. Exclusions were made for persons who received COVID-19 vaccine doses ≤30 days before infection or who had infections ≤30 days before vaccination.

Cardiac complications were rare after SARS-CoV-2 infection or mRNA COVID-19 vaccination. However, the risks for these complications were higher after infection than after vaccination among males and females in all age groups. These findings provide important context for balancing risks and benefits of mRNA COVID-19 vaccination among eligible persons ≥5 years.

**Summary**

**What is already known about this topic?**

Studies have found an increased risk for cardiac complications after SARS-CoV-2 infection and mRNA COVID-19 vaccination, but few have compared these risks.

**What is added by this report?**

Data from 40 health care systems participating in a large network found that the risk for cardiac complications was significantly higher after SARS-CoV-2 infection than after mRNA COVID-19 vaccination for both males and females in all age groups.

**What are the implications for public health practice?**

These findings support continued use of recommended mRNA COVID-19 vaccines among all eligible persons aged ≥5 years.

## Footnotes

* The 40 PCORnet sites were AdventHealth, Allina Health, Children’s Hospital Colorado, Cincinnati Children’s Hospital, Columbia Health, Duke University, Fenway Health, Health Choice Network, Johns Hopkins University, Lurie Children’s Hospital, Medical College of Wisconsin, Medical University of South Carolina, Montefiore Medical Center, Mount Sinai Health System, Nationwide Children’s Hospital, Nemours Children’s Hospital, New York University Langone Medical Center, Northwestern University, OCHIN, Inc., Ochsner Health System, Ohio State University, Orlando Health System, Penn State College of Medicine and Penn State Health Milton S. Hershey Medical Center, Seattle Children’s Hospital, Temple University, University of Florida Health, University of Iowa Healthcare, University of Kansas, University Medical Center New Orleans, University of Miami, University of Michigan, University of Missouri Health Care, University of Nebraska, University of North Carolina, University of Pittsburgh Medical Center, University of Texas Southwest Medical Center, University of Utah, Vanderbilt University Medical Center, Wake Forest Baptist Health, and Weill Cornell Medicine. These sites represent academic and community hospitals that serve patients who are self-pay or have public or private insurance.

† The first dose cohort included persons who had either the first of 2 doses ≥20 days before a second dose or a specific code for a first dose; the second dose cohort included persons who had either the second of 2 doses ≥20 days after a first dose or a specific code for a second dose. The unspecified dose cohort included persons who had only one code for an mRNA COVID-19 vaccination that was not specified as a first or second dose. The any dose cohort was the combination of the first, second, and unspecified dose cohorts; this cohort included all doses captured, with duplication of persons who received 2 doses. Vaccination and infection exclusions were provided before but not after exposures; thus, persons who had an infection soon after a vaccination would still be included in the vaccination cohort or vice versa. The cohorts were not mutually exclusive; persons vaccinated and infected could be in both vaccination and infection cohorts. However, because the outcomes were assessed in short time periods after index dates, overlap in outcomes was unlikely, unless an outcome was experienced more than once.

§ Myocarditis was defined as presence of ICD-10-CM codes B33.22, I40, I40.0, I40.1, I40.8, I40.9, or I51.4. Pericarditis was defined as presence of ICD-10-CM codes B33.23, I30, I30.0, I30.1, I30.8, I30.9, or I31.9. MIS was defined as presence of ICD-10-CM code M35.81.

¶ MIS often occurs in the absence of prior positive SARS-CoV-2 test results; these cases were not captured in the infection cohorts.

** 45 C.F.R. part 46, 21 C.F.R. part 56; 42 U.S.C. Sect. 241(d); 5 U.S.C. Sect. 552a; 44 U.S.C. Sect. Sect. 3501 et seq.

†† In the first and second dose vaccine cohorts, 27% of persons received Moderna and 73% received Pfizer-BioNTech. In the unspecified dose cohort, 36% received Moderna and 64% Pfizer-BioNTech, and in the any dose cohort, 29% received Moderna and 71% Pfizer-BioNTech.

§§ https://covid.cdc.gov/covid-data-tracker/#vaccinations (Accessed March 29, 2022).

¶¶ If patients who received a SARS-CoV-2–positive test result at a health care system were more likely to return to the same health care system for myocarditis, pericarditis, or MIS treatment than were patients who had their mRNA COVID-19 vaccination documented at the health care system, then the underascertainment of outcomes might be higher in the vaccination cohorts, introducing bias away from the null. This scenario might occur if a person was more likely to visit a tertiary care referral center participating in this study if they were more severely ill with a cardiac complication after SARS-CoV-2 infection than a perhaps mild cardiac complication after COVID-19 vaccination. However, if the cardiac complications were more commonly linked to vaccination than infection in the EHR, bias would be toward the null. This scenario might occur if clinicians were more likely to document an mRNA COVID-19 vaccination in the EHR if a cardiac complication was noted after vaccination than if the cardiac complication occurred after SARS-CoV-2 infection.
